# Vitamin D Supplementation Improves Quality of Life and Physical Performance in Osteoarthritis Patients

**DOI:** 10.3390/nu9080799

**Published:** 2017-07-26

**Authors:** Pacharee Manoy, Pongsak Yuktanandana, Aree Tanavalee, Wilai Anomasiri, Srihatach Ngarmukos, Thanathep Tanpowpong, Sittisak Honsawek

**Affiliations:** 1 Program in Medical Sciences, Faculty of Medicine, Chulalongkorn University, King Chulalongkorn Memorial Hospital, Thai Red Cross Society, 1873 Rama IV Road, Pathumwan, Bangkok 10330, Thailand; pachareeman@gmail.com; 2 Vinai Parkpian Orthopaedic Research Center, Department of Orthopaedics, Faculty of Medicine, Chulalongkorn University, King Chulalongkorn Memorial Hospital, Thai Red Cross Society, 1873 Rama IV Road, Pathumwan, Bangkok 10330, Thailand; ypongsak@gmail.com (P.Y.); areetana@hotmail.com (A.T.); srihatach@hotmail.com (S.N.); thanathep1@yahoo.com (T.T.); 3 Department of Biochemistry, Faculty of Medicine, Chulalongkorn University, King Chulalongkorn Memorial Hospital, Thai Red Cross Society, 1873 Rama IV Road, Pathumwan, Bangkok 10330, Thailand; awilaiano@gmail.com

**Keywords:** vitamin D_2_ supplementation, osteoarthritis, muscle strength, physical performance

## Abstract

(1) *Background*: Lower levels of serum 25-hydroxyvitamin D (25(OH)D) are common in osteoarthritis (OA) patients. However, the effect of vitamin D supplementation on muscle strength and physical performance remains unclear. This study will investigate the effects of vitamin D_2_ supplementation on muscle strength and physical performance in knee OA patients; (2) *Methods*: One hundred and seventy-five primary knee OA patients with low levels of serum 25(OH)D (<30 ng/mL) received 40,000 IU vitamin D_2_ (ergocalciferol) per week for six months. Body composition, muscle strength, physical performance, serum 25(OH)D level, leptin, interlukin-6 (IL-6), parathyroid hormone (PTH), protein carbonyl, and metabolic profile were analyzed; (3) *Results*: Baseline mean serum 25(OH)D levels in knee OA patients was 20.73 ng/mL. Regarding baseline vitamin D status, 58.90% of patients had vitamin D insufficiency, and 41.10% had vitamin D deficiency. After vitamin D_2_ supplementation for six months, mean serum 25(OH)D level was 32.14 ng/mL. For post-supplementation vitamin D status, 57.10% of patients had vitamin D sufficiency and 42.90% had vitamin D insufficiency. From baseline to six months, there was a significant increase in mean serum 25(OH)D level (*p* < 0.001), while mean LDL cholesterol (*p* = 0.001), protein carbonyl (*p* = 0.04), and PTH (*p* = 0.005) all significantly decreased. Patient quality of life (SF-12) and pain (visual analog scale, VAS) both improved significantly from baseline to the six-month time point (*p* = 0.005 and *p* = 0.002, respectively). Knee OA patients demonstrated significant improvement grip strength and physical performance measurements after vitamin D_2_ supplementation (*p* < 0.05); (4) *Conclusions*: Vitamin D_2_ supplementation for six months reduced oxidative protein damage, decreased pain (VAS), improved quality of life, and improved grip strength and physical performance in osteoarthritis patients.

## 1. Introduction

Osteoarthritis (OA) is the most common cause of musculoskeletal disability and pain worldwide. OA is characterized by the degradation of articular cartilage, including changes in subchondral bone, osteophyte formation, joint space narrowing, and synovial inflammation [1]. Symptoms of disease include joint pain, knee muscle wasting, and decreased range of motion, all of which lead to severe pain and disability in later life [2]. There are many risk factors that lead to early structural changes of the knee among healthy individuals. Vitamin D deficiency may play a role in the pathogenesis of OA on a clinical level [3]. Vitamin D deficiency has been associated with poor physical performance in the elderly [4], and 63% of primary knee OA patients were found to have low vitamin D status [5]. Accordingly, lower levels of 25-hydroxyvitamin D (25(OH)D) were associated with greater knee pain, increased progression of radiographic OA [6], and poor quadriceps function [7].

Vitamin D supplementation is an alternative treatment in elderly people who are at greater risk of vitamin D deficiency and tend to have poor physical function. Several studies have reported that vitamin D supplementation increases muscle strength, improves physical function, and decreases risk of falls among older people with low level of serum vitamin D [8,9,10]. However, other previous studies reported that vitamin D supplementation did not improve muscle strength or physical function [11,12,13]. Using the Western Ontario and McMaster Universities (WOMAC) Osteoarthritis Index and visual analog scale (VAS) assessment, the effects of vitamin D supplementation were reported to decrease pain and improve knee function in OA patients [5]. In contrast, another previous study reported no significant positive effect of vitamin D supplementation on the prevention of tibial cartilage loss or improvement in WOMAC knee pain [14].

Although vitamin D_2_ (ergocalciferol) and vitamin D_3_ (cholecalciferol) are available over the counter as dietary supplements and do not require a prescription, ergocalciferol is the only therapeutic agent that is a first-line drug (category A) for vitamin D deficiency used in the hospitals and public health sectors in Thailand. Given this disparity in the previous finding regarding vitamin D supplementation in Thailand, vitamin D_2_ (ergocalciferol) was used in this study for the investigation of the role of vitamin D supplementation on muscle strength and physical performance in knee OA patients with vitamin D insufficiency and deficiency. The secondary objective of this study was to assess the possible benefits of vitamin D supplementation on metabolic risk factors, levels of inflammation, adipokine, and oxidative stress.

## 2. Materials and Methods

### 2.1. Study Design and Participants

This controlled before–after study was conducted at the outpatient clinic of the Department of Orthopedics at King Chulalongkorn Memorial Hospital during a February–December 2015 study period. Two hundred and thirty-eight patients with knee OA agreed to participate. All had knee OA based on the criteria of American College of Rheumatology classification. The inclusion criteria were that the participants had symptomatic knee OA (Kellgren–Lawrence grading of ≤2) and low vitamin D status (25(OH)D < 30 ng/mL). The diagnosis of osteoarthritis is based primarily on patient history, physical examination, and radiographic findings. Exclusion criteria included history of knee surgery, primary hyperparathyroidism, rheumatoid or other inflammatory arthritis (i.e., septic arthritis, gout), neurological condition (i.e., Parkinson’s disease, previous stroke), or inability to perform physical activity.

**Figure 1:**
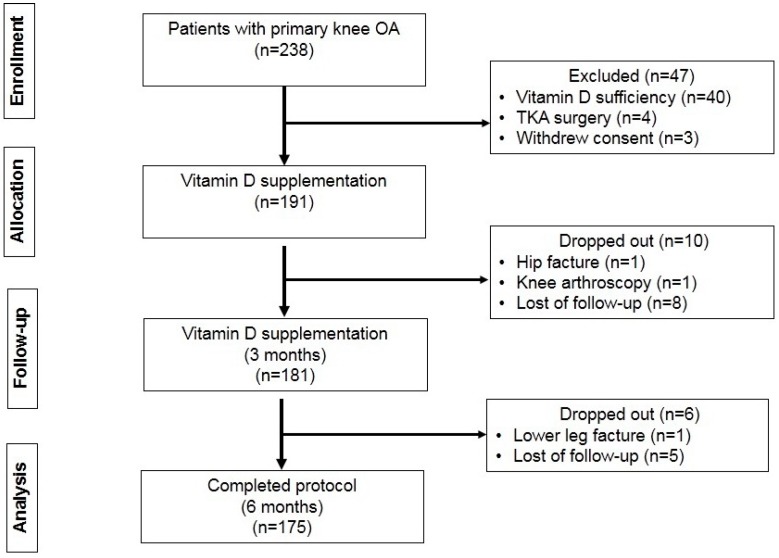
Flowchart of the study protocol.

One hundred and ninety-one patients met the study criteria and were included. Sixteen of the included patients were not included in the final analysis for the following reasons: 13 patients were lost of follow-up, two sustained fracture (hip and lower leg–one each), and one patient had knee arthroscopy. A total of 175 knee OA patients completed the study protocol and were analyzed (Figure 1).

The study protocol was approved by the Institutional Review Board of the Faculty of Medicine at Chulalongkorn University (IRB approval No. 512/57). Written informed consent was obtained from all participants prior to their participation in the study.

### 2.2. Interventions

The Endocrine Society guidelines suggest that 50,000 IU of vitamin D_2_ taken once a week for eight weeks is necessary to achieve the levels of serum 25(OH)D consistently above 30 ng/mL in adults [15]. In Thailand, the only available form of vitamin D_2_ (egocalciferol) is in the form of 20,000 IU/capsule. Therefore, each subject was asked to take 40,000 IU of vitamin D_2_ (two capsules of 20,000 IU ergocalciferol; the British Dispensary, Bangkok, Thailand) per week for six months in order to evaluate the effect of vitamin D supplementation on muscle function and biochemical markers.

### 2.3. General Information

All participants were evaluated for knee pain using WOMAC and VAS evaluation instruments. VAS score is based on a 0–10 point scale, with a higher score indicating a higher level of pain. The participants were asked to put a mark on the line indicating their pain intensity at the present time in response to the following question: “If “0” is “no pain” and “10” is “the worst pain”, where is your average pain intensity now on the visual analog score (VAS)?” [16]. Total WOMAC score represented the sum of three subscales, including pain, stiffness, and physical function. A higher WOMAC score indicates worse pain, more stiffness, and increased functional limitations. A Thai version of the Short-Form Health Survey (SF-12) evaluated health-related quality of life, including physical health composite score (PCS) and mental health composite score (MCS), both of which range from 0 to 100, with a higher score indicating better quality of life and well-being. Physical activity was evaluated using the Thai version of the Physical Activity Questionnaire for Elderly Japanese (PAQ-EJ). PAQ-EJ physical activity measurements mirror patterns of daily activity among elderly Thai and other elderly Asian people [17]. PAQ-EJ scores were converted to metabolic equivalent of task (MET) hours per week (MET h/week) [18].

### 2.4. Anthropometric and Body Composition Measurements

Height, weight, and waist circumference (WC) were determined using standard measurement techniques. Body mass index (BMI) was calculated by dividing weight (kg) by the square of height (m^2^). Appendicular skeletal muscle mass (ASM), percentage of total fat mass, fat mass (FM), and fat-free mass (FFM) were assessed using bioelectrical impedance analysis (BIA) (BC-418 Segmental Body Composition Analyzer; Tanita Corporation, Tokyo, Japan). ASM was estimated as the sum of the skeletal muscle mass of the arms and legs in kilograms. The appendicular skeletal muscle mass index (ASMI) was calculated as ASM divided by squared height. Skeletal muscle index (SMI) was calculated as percentage of appendicular skeletal muscle mass (ASM) divided by body weight (%).

### 2.5. Muscle Strength and Physical Performance

At baseline, three months, and six months, muscle strength and physical performance were measured by physical therapists. Grip strength was assessed by grip strength dynamometer (Takei Scientific Instruments Co. Ltd., Tokyo, Japan) (kilograms) [19]. Knee extension force was measured by a handheld MicroFET 2 dynamometer (Hoggan Scientific LLC, Salt Lake City, UT, USA) (Newtons). The participant sat on the treatment table with knees flexed 90° and the dynamometer was applied to the anterior part of the leg, 5 cm above the transmalleolar axis and perpendicular to the tibial crest. The participant raised their lower legs up and held against a maximum persistent force position (5 s) applied by a physical therapist [20]. Four tests were used to evaluate physical performance. The first test was the 4-m gait speed test, which measures the time needed to walk four meters, calculated as meters per second [19]. The second test was the Timed Up and Go Test (TUGT), which measured the time needed to stand up from a chair, walk three meters, and return to the chair and sit down (seconds) [21]. The third test was the five times sit-to-stand test (STS), which recorded the time needed to perform five repeated chair stands without the use of arms (seconds) [22]. The last of the four tests was the six-minute walk test (6MWT), which measured the distance a patient could walk in six minutes (in meters) [21].

### 2.6. Biochemical Analysis

At baseline and six months, fasting early-morning venous blood was collected and centrifuged, with serum and plasma samples stored at −70 °C until use. Fasting blood glucose (FBG), lipid profile, calcium, phosphorus, and high-sensitivity C-reactive protein (hs-CRP) were measured using an autoanalyzer (Architech 16,000 Analyzer, Abbott Diagnostics, Irving, TX, USA). Serum levels of leptin and interlukin-6 (IL-6) were determined by enzyme-linked immunosorbent assay using kits from R&D Systems, Minneapolis, MN, USA and BioLegend, San Diego, CA, USA, respectively. Plasma level of protein carbonyl was assessed by spectrophotometer, according to the method of Castegna et al., 2003 [23] Serum 25(OH)D level was measured by chemiluminescent immunoassay (DiaSorin, Inc., Stillwater, MN, USA). PTH and insulin were determined by electrochemiluminescence method (Roche Diagnostics GmbH, Mannheim, Germany). Insulin resistance was calculated using homeostasis model assessment (HOMA-IR) using the following formula: fasting serum insulin (µU/mL) × fasting plasma glucose (mg/dL)/405. Vitamin D deficiency was defined as <20 ng/mL, vitamin D insufficiency as 20 -< 30 ng/mL, and vitamin D sufficiency as ≥30 ng/mL.

### 2.7. Statistical Analysis

Data were analyzed using SPSS Statistics version 22 (SPSS, Inc., Chicago, IL, USA). Comparison of baseline vs. post-vitamin D_2_ supplementation data was performed by paired *t*-test. One-way repeated-measurement ANOVA was used to test the time differences in muscle strength and physical performance. Correlation between variables was tested by Spearman’s rank correlation coefficient (*r*). Data are summarized as mean ± standard error of the mean (SEM). A *p*-value less than 0.05 for differences and correlations was considered to be statistically significant.

## 3. Results

### 3.1. Effects on Body Composition, Pain, Quality of Life and Physical Activity

A total of 175 participants (158 females and 17 males) with a mean age of 64.58 ± 0.55 years. After vitamin D_2_ supplementation, weight, percent of fat, fat mass, and visceral fat were all significantly decreased, when compared to baseline levels (*p* < 0.05) (Table 1).

WOMAC and PAQ-EJ scores did not change significantly between baseline and six months. However, VAS decreased significantly after treatment (*p* = 0.002) and the PCS of SF-12 improved significantly after supplementation treatment (*p* = 0.005). 

### 3.2. Effects on Metabolic Risk Factors

HDL cholesterol levels increased after treatment, but the change was not statistically significant. LDL cholesterol levels significantly decreased after vitamin D_2_ supplementation (*p* = 0.001). FBG, Insulin, HOMA-IR, and blood pressure did not change between baseline and six months, as shown in Table 1.

### 3.3. Effects on Vitamin D and PTH Status

**Figure 2:**
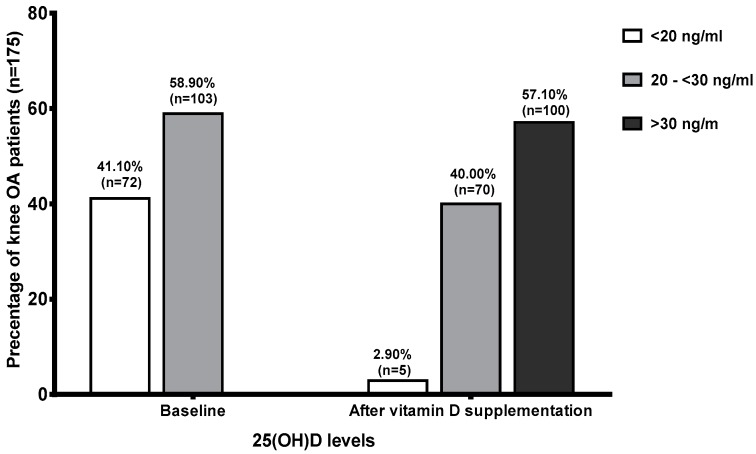
Vitamin D status in knee OA patients at baseline and after vitamin D_2_ supplementation.

At baseline, the mean serum 25(OH)D level in knee OA patients was 20.73 ± 0.36 ng/mL. Seventy-two participants (41.10%) had vitamin D deficiency, and 103 patients (58.90%) had vitamin D insufficiency. After 40,000 IU of vitamin D_2_ supplementation per week for six months, there was a statistically significant increase in mean serum 25(OH)D level to 32.14 ± 0.59 ng/mL (*p* < 0.001) (Table 1). One hundred (57.10%) knee OA participants that previously had either vitamin D insufficiency or deficiency at baseline achieved serum 25(OH)D concentration above 30 ng/mL (28 with baseline deficiency and 72 with baseline insufficiency). After supplementation and at the six-month time point, 70 knee OA participants (40.00%) had vitamin D insufficiency, and only five patients (2.90%) had vitamin D deficiency (Figure 2). During treatment, levels of serum calcium increased significantly (*p* < 0.05), three OA patients (1.71%) developed hypercalcemia (Ca > 10.5 mg/dL) and PTH decreased significantly (*p* < 0.05) after vitamin D_2_ supplementation.

### 3.4. Effects on Inflammation, Adipokine and Oxidative Stress

Levels of hs-CRP, IL-6 and leptin were not different (*p* > 0.05), but protein carbonyls concentration was significantly decreased between baseline and after vitamin D_2_ supplementation (*p* = 0.04).

### 3.5. Effects on Muscle Strength and Physical Performance

Dominant grip strength (*p* = 0.01) and overall physical performance, such as gait speed (*p* < 0.001), TUGT (*p* < 0.001), STS (*p* < 0.001), and 6MWT (*p* < 0.001), significantly improved after vitamin D_2_ supplementation, but there were no significant difference observed for non-dominant grip strength and knee extension force between baseline and post-treatment (*p* > 0.05) are presented in Table 2.

### 3.6. Association of 25(OH)D, Biochemical Markers and Body Composition

**Figure 3:**
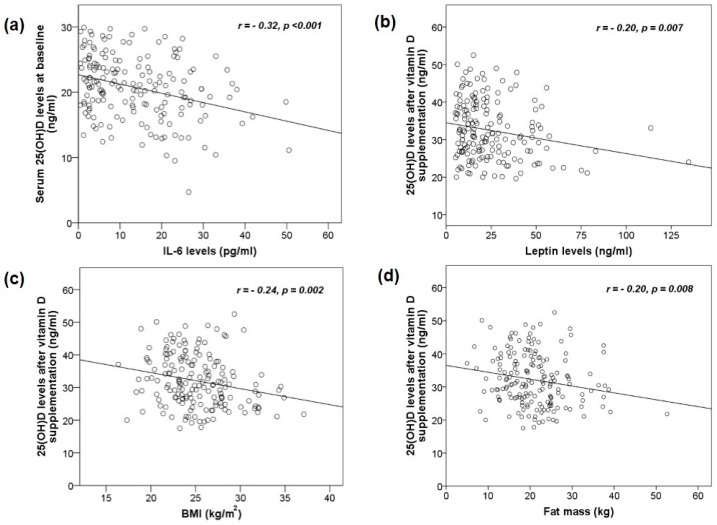
A negative association between 25(OH)D levels and biomarkers (**a**) IL-6 levels at baseline; (**b**) leptin levels after vitamin D supplementation. The association between of vitamin D levels and body composition after vitamin D supplementation; (**c**) BMI and (**d**) fat mass were negatively associated with 25(OH)D levels.

We found a negative correlation between 25(OH)D and IL-6 at baseline (*r* = −0.32, *p* < 0.001). After vitamin D_2_ supplementation, our results showed that 25(OH)D level was negatively correlated with leptin (*r* = −0.20, *p* = 0.007), BMI (*r* = −0.24, *p* = 0.002) and fat mass (*r* = −0.20, *p* = 0.008) are shown in Figure 3. Correlations between 25(OH)D level, muscle strength, and physical performance were not significantly different between baseline and after treatment (*p* > 0.05).

## 4. Discussion

The objective of this study was to determine whether vitamin D supplementation could improve muscle strength and physical performance in knee OA patients with low vitamin D status. The results showed that knee OA with vitamin D_2_ supplementation improved grip strength and physical performance, but did not improve knee extension force. We also found that vitamin D supplementation reduced oxidative protein damage, reduced pain, and improved quality of life.

Six months after supplementation of 40,000 IU of vitamin D_2_ per week, 57% of patients achieved vitamin D sufficiency, whereas 40% and 3% had vitamin D insufficiency and deficiency, respectively. Generally, the source of vitamin D supplementation from diet and dietary supplements are ergocalciferol (vitamin D_2_) and cholecalciferol (vitamin D_3_), which are inactive forms of vitamin D. Vitamin D_2_ are found plant and yeast irradiation, whereas the sources of vitamin D_3_ are oily fish and meat [24]. Similarly, two types of vitamin D supplementation are available for over-the-counter purchase. In Thailand, ergocalciferol is used to treat vitamin D deficiency as the first-line therapeutic drug. However, some evidence suggests that vitamin D_2_ should not be regarded as equivalent to vitamin D_3_ for maintaining the concentration of 25(OH)D [25]. Serum 25(OH)D_2_ has a lower affinity for vitamin D-binding protein (DBP), and the serum half-life of 25(OH)D_2_ is shorter than 25(OH)D_3_ [26]. There is possibly a higher affinity of hepatic 25-hydroxylase for vitamin D_3_ than for vitamin D_2_ [27]. The results of our study have demonstrated that 40,000 IU of vitamin D_2_ per week was able to enhance 25(OH)D levels to achieve vitamin D sufficiency in only 57% of participants. In fact, other factors may influence the increment of vitamin D levels, such as dietary vitamin D intake and exposure to the sunlight, which were not included in this study. According to the experimental design in this study, only one group of the population was deployed to study the effect of vitamin D supplementation between before and after supplementation. We believed that they would have been exposed to an equivalent amount of sunlight and consumed vitamin D-containing food in similar amounts before and after supplementation due to their daily behaviors.

Vitamin D_2_ supplementation also affected calcium and PTH levels. We found that serum Ca levels increased and PTH levels decreased significantly after supplementation. Only 1.71% (*n* = 3) of cases had mild hypercalcemia after vitamin D_2_ supplementation. Pietras et al. reported no incidents of vitamin D toxicity and normal levels of serum calcium in patients who were treated with 50,000 IU of vitamin D_2_ every other week for up to six years [28]. Del Valle et al. studied a high-dose ergocalciferol 72,000 IU/week for 12 weeks and maintenance therapy 24,000 IU/week during 36 weeks in hemodialysis patients. They found that only 1.8% had hypercalcemia [29]. However, blood calcium levels are not a good reflection of calcium status, whereas urinary calcium excretion determines the risks of vitamin D treatment for excessive calcium absorption. Consequently, the results revealed that PTH levels significantly decreased after treatment. The previous study reported that low vitamin D status was associated with elevated bone turnover by increasing PTH levels [30]. Moreover, high levels of PTH are related with the risk of fall, fracture, and poorer outcomes in terms of frailty [31]. PTH action stimulates the transformation of pro-osteoclasts into mature osteoclasts, which leads to increasing bone turnover [32]. Consequently, optimal vitamin D levels may help to reduce the risk of fall, fracture, and osteoporosis.

Body composition, including weight, percentage of fat, fat mass and visceral fat rating, all decreased significantly after vitamin D_2_ supplementation compared with their baseline values, but skeletal muscle mass did not change. Our results showed that the participants lost weight, which might be due to change in their lifestyles, and had significantly improved physical function according to increasing physical health composite scores (PCS) of SF-12, while physical activity assessments from PAQ-EJ did not differ. Moreover, we also observed weak negative association between both 25(OH)D and BMI and 25(OH)D and fat mass after vitamin D supplementation. Consistent with our result, Lagari et al. reported that higher fat mass was associated with lower vitamin D status [33]. Therefore, patients with a higher BMI or obesity may experience slower increases in serum vitamin D level than people with normal or thin body composition. This suggests that higher doses of vitamin D supplementation and longer treatment times may be needed in knee OA patients with higher BMI or obesity.

Self-reported pain and health-related quality of life showed improvement after vitamin D supplementation according to results obtained from VAS and the PCS of SF-12 questionnaires. However, WOMAS score is not relevant. The previous study reported that WOMAC and VAS decreased significantly after vitamin D supplementation [5]. In contrast, other studies reported that vitamin D supplementation did not reduce knee pain, cartilage volume loss, or improve physical function [14,34]. Actually, VAS assessed severity of pain from the patient’s perspective at the moment of assessment, and the pain VAS is a single-item scale. The WOMAC score used in the evaluation of knee OA consists of three subscales such as pain, stiffness and physical function (the questions cover everyday activities). Therefore, the effect of vitamin D supplementation on VAS may not be a good reflection of pain during daily activities.

The effect of vitamin D supplementation on metabolic risk factors presented a significant reduction in LDL-cholesterol. The participants had lost weight, decreased fat percentages, and lower fat mass and visceral fat ratings, which may have result in the reduced LDL-cholesterol levels in this study. The previous studies have shown a significant reduction in LDL-cholesterol levels after vitamin D supplementation [35,36]. The effects of vitamin D increase level of intestinal calcium intake, while calcium may reduce fatty acid absorption due to the formation of insoluble calcium–fatty complexes in the gut. Therefore, serum levels of LDL-cholesterol would be decreased by the reduced absorption of saturated fatty acids [37]. However, vitamin D supplementation did not improve lipid profiles in obese individuals [38,39].

In regards to the relationship between vitamin D, inflammation, and adipokine, the results demonstrated a weak negative association of 25(OH)D with IL-6 and leptin. These results were consistent with a previous report that vitamin D deficiency was associated with more pro-inflammatory cytokines as compared with insufficiency or sufficiency status in elderly adults [40]. Moreover, the previous studies reported a negative association between serum 25(OH)D and leptin concentrations [41,42].

In addition, our data showed that vitamin D supplementation reduced oxidative protein damage by decreasing levels of protein carbonyl. Protein carbonyl was used as the biomarker of oxidative damage, since it leads to cellular dysfunction and a decline in muscle function [43,44]. It is the mechanism involved in the direct oxidation of amino acids such as lysine, arginine, histidine, proline, glutamic acid, and threonine, or by the binding of aldehydes produced from lipid peroxidation [23]. Carbonyl stress can modify protein function and cause DNA damage through stimulating pro-inflammatory signaling (nuclear factor-ĸB: NF-ĸB & p38), tissue remodeling, muscle dysfunction, [45] and the pathogenesis of sarcopenia [46]. Vitamin D may be regarded as an antioxidant in which 1,25-dihydroxyvitamin D (1,25(OH)_2_D) binding to the vitamin D receptor (VDR) and the retinoid X receptor (RXR) interact with various nuclear co-activators that regulate gene transcription. It may reduce reactive oxygen species formation by the suppression of the gene expression of NADPH oxidase, and induce the expression of antioxidant genes [47]. Moreover, 1,25(OH)_2_D has been demonstrated to suppress the production of pro-inflammatory cytokines, such as IL-6 and tumor necrosis factor-α (TNF-α), as well as reduce the expression of NF-ĸB and p38 [48]. These findings suggest that high levels of vitamin D after supplementation may reduce the amounts of reactive oxygen species produced by damaging proteins.

Regarding muscle strength and physical performance, we found that knee OA patients significantly improved grip strength and physical performance, but did not improve knee extension force. In this aspect, our results are consistent with the findings of several previous studies. Zhu et al. reported that hip muscle strength and TUGT improved significantly after 1000 IU/day vitamin D_2_ supplementation for one year in older women with vitamin D insufficiency [49]. Lagari et al. reported that vitamin D supplementation might be most beneficial in older populations with poor physical function [33]. Sato et al. found that the mean of type II muscle fiber diameter and percentage of type II fibers increased significantly after 1000 IU/day vitamin D_2_ treatment over two years in elderly patients with post-stroke hemiplegia [50]. Ceglia et al. reported that intramyonuclear VDR concentration increased 30% and total (type I and II) muscle fiber size increased 10% after vitamin D supplementation in mobility-limited elderly women [51]. However, some studies have reported that vitamin D supplementation did not improve muscle strength or physical function. Kenny et al. found that vitamin D supplementation did not improve muscle strength or physical performance in a group of healthy community-dwelling older men [11]. These conflicting findings may be attributed to differences in populations, disease advancement, or measurements applied, or to incomplete control of confounding variables. Nonetheless, conclusions should be drawn with caution on whether the characteristics of studied participants or the dose of vitamin D used are of significance, as these studies were heterogeneous with regards to most aspects. Various outcome measures have been documented by different investigators and even in the case of measurements of similar characteristics, different methods have been applied, making it difficult to compare studies directly.

A strength of this study is the finding that a high dose and a long-term intervention of vitamin D_2_ supplementation was effective in raising 25(OH)D concentrations. It is possible that achieved serum 25(OH)D levels may improve muscle function by increasing muscle strength and physical performance in knee OA patients. Higher serum 25(OH)D concentrations may be essential in skeletal muscle, particularly for the elderly with limited mobility [33,50,51]. On the other hand, increasing 25(OH)D levels in healthy populations do not relate to any improvement of muscle function [11]. Therefore, patients with impaired mobility may be more sensitive to the improvement in physical functioning by vitamin D supplementation. Previous studies indicated that vitamin D supplementation in the elderly with vitamin D insufficiency reduced an atrophy of type II muscle fiber [50] and increased the size of type I and II muscle fiber, as well as VDR concentration [51]. Actually, knee OA patients with poor muscle function and vitamin D deficiency may be the most likely to benefit from vitamin D supplementation.

This study has several mentionable limitations. First, the controlled before–after design of this study did not include a control group. The lack of randomization, and our decision not to evaluate the sensitivity of drug effect, potentially weaken our findings relative to the therapeutic effect of vitamin D supplementation. Second, the sample size was small and the proportion of men was low, both of which prevented us from establishing the clinical relevance, particularly regarding changes in muscle strength. Third, we assayed markers of oxidative damage using plasma protein carbonyls that were not directly measured in skeletal muscle. Finally, 8.37% of patients were lost to follow-up. While this rate is higher than can be considered ideal, the loss to follow-up rate in the present study was lower than loss to follow-up rates reported from other studies.

## 5. Conclusions

In conclusion, our results suggest that 40,000 IU of vitamin D_2_ supplementation reduced oxidative protein damage, improved quality of life, and improved grip strength and physical performance. It remains unclear whether vitamin D supplementation relates to musculoskeletal pain or not. Accordingly, vitamin D treatment decreases current pain using VAS, but does not reduce pain during physical activity, as determined by WOMAC score. Nevertheless, vitamin D supplementation is a safe and inexpensive way to improve muscle strength and physical function in this population. Based on these findings, we recommend vitamin D supplementation in knee OA patients that have poor physical function.

## Figures and Tables

**Table 1:** Demographic data before and after vitamin D_2_ supplementation in six months.

	Vitamin D_2_ Supplementation (*n* = 175)			*p*-Value
	Mean ± SEM		Mean Difference (95% CI)	
	Baseline	6 Months		
**Gender** (F/M)	158:17	158:17		
**Age** (years)	64.58 ± 0.55	64.58 ± 0.55		
**Body composition**				
Waist circumference (cm)	87.87 ± 0.73	87.82 ± 0.71	−0.05 (−0.66 to 0.56)	0.87
Weight (kg)	62.38 ± 0.89	61.70 ± 0.88	−0.68 (−1.27 to −0.09)	0.02
BMI (kg/m^2^)	25.63 ± 0.30	25.41 ± 0.30	−0.22 (−0.48 to 0.04)	0.09
Percentage of fat (%)	35.42 ± 0.52	33.28 ± 0.52	−2.14 (−2.80 to −1.47)	<0.001
Fat mass (kg)	22.66 ± 0.59	20.93 ± 0.54	−1.72 (−2.30 to −1.14)	<0.001
Visceral fat rating	9.46 ± 0.29	9.03 ± 0.24	−0.43 (−0.72 to −0.13)	0.004
ASM (kg)	17.58 ± 0.28	17.50 ± 0.27	−0.08 (−0.20 to 0.03)	0.15
ASMI (kg/m^2^)	7.20 ± 0.08	7.18 ± 0.08	−0.03 (−0.08 to 0.01)	0.18
SMI (%)	28.37 ± 0.27	28.52 ± 0.25	0.14 (−0.15 to 0.44)	0.33
**VAS** (0–10)	3.96 ± 0.17	3.44 ± 0.17	−0.51 (−0.83 to −0.19)	0.002
**WOMAC**				
Pain (0–10)	2.45 ± 0.15	2.59 ± 0.15	0.14 (−0.15 to 0.44)	0.33
Stiffness (0–10)	2.56 ± 0.18	2.26 ± 0.16	−0.29 (−0.62 to 0.03)	0.08
Physical disability (0–10)	2.90 ± 0.15	2.76 ± 0.15	−0.14 (−0.41 to 0.13)	0.31
Total score (0–10)	2.80 ± 0.13	2.78 ± 0.13	−0.01 (−0.08 to 0.06)	0.75
**SF-12**				
PCS (0–100)	38.26 ± 0.65	40.24 ± 0.67	1.98 (0.60 to 3.36)	0.005
MCS (0–100)	50.00 ± 0.70	49.57 ± 0.66	−0.42 (−1.82 to 0.97)	0.54
**Physical activity**				
PAQ-EJ (MET hours/week)	52.28 ± 2.83	53.29 ± 3.08	1.00 (−5.11 to 7.13)	0.74
**Metabolic risk factors**				
FBG (mg/dL)	98.06 ± 1.30	98.49 ± 1.56	0.42 (−2.26 to 3.12)	0.75
Insulin (µU/mL)	5.32 ± 0.41	5.99 ± 0.41	0.66 (−0.20 to 1.53)	0.13
HOMA-IR	1.34 ± 0.11	1.55 ± 0.13	0.20 (−0.06 to 0.46)	0.13
Total cholesterol (mg/dL)	211.94 ± 2.93	212.90 ± 3.14	0.95 (−4.35 to 6.26)	0.72
HDL cholesterol (mg/dL)	55.30 ± 1.00	57.40 ± 1.28	2.09 (−0.03 to 4.23)	0.05
Triglycerides (mg/dL)	126.34 ± 4.16	123.70 ± 4.63	−2.63 (−10.32 to 5.04)	0.49
LDL (mg/dL)	135.42 ± 2.76	127.64 ± 2.78	−7.77 (−12.43 to −3.12)	0.001
SBP (mmHg)	131.02 ± 0.77	131.00 ± 0.81	−0.77 (−0.66 to 0.60)	0.93
DBP (mmHg)	78.57 ± 0.51	78.25 ± 0.55	−0.81 (−0.80 to 0.16)	0.19
**Biochemical markers**				
25(OH)D (ng/mL)	20.73 ± 0.36	32.14 ± 0.59	11.41(10.27 to 12.54)	<0.001
Calcium (mg/dL)	9.25 ± 0.03	9.34 ± 0.04	0.09 (0.006 to 0.18)	0.03
Phosphorus (mg/dL)	3.62 ± 0.03	3.69 ± 0.03	0.06 (−0.01 to 0.13)	0.10
PTH (pg/mL)	53.20 ± 1.72	46.63 ± 2.21	−6.57 (−11.08 to −2.05)	0.005
hs-CRP (mg/dL)	1.97 ± 0.20	2.61 ± 0.34	0.64 (−0.06 to 1.35)	0.07
IL-6 (pg/mL)	20.59 ± 4.52	22.37 ± 2.32	1.78 (−5.75 to 9.31)	0.64
Leptin (ng/mL)	25.93 ± 1.57	24.68 ± 1.45	−1.24 (−3.89 to 1.39)	0.35
Protein carbonyls (nmol/mg)	0.79 ± 0.04	0.70 ± 0.03	−0.08 (−0.16 to −0.003)	0.04

Abbreviations: F: female, M: male, BMI: body mass index, ASM: appendicular skeletal muscle mass, ASMI: appendicular skeletal muscle mass index, SMI: skeletal muscle index, VAS: visual analogue scale, WOMAC: Western Ontario and McMaster Universities Osteoarthritis Index, SF-12: 12-Item short form health survey, PCS: physical health composite scores, MCS: mental health composite scores, PAQ-EJ: physical activity questionnaire for elderly Japanese in Thai version, MET: metabolic equivalent of task, FBG: fasting blood glucose, HOMA-IR: homeostatic model assessment of insulin resistance, HDL-cholesterol: high-density lipoprotein cholesterol, LDL-cholesterol: low-density lipoprotein cholesterol, SBP: systolic blood pressure and DBP: diastolic blood pressure, 25(OH)D, 25-hydroxyvitamin D and PTH: parathyroid hormone.

**Table 2:** Muscle strength and physical performance at baseline, three months and six months after vitamin D_2_ supplementation.

	Vitamin D_2_ Supplementation (*n* = 175)			*p*-Value
	Mean ± SEM			
	Baseline	3 Months	6 Months	
**Grip strength (kg)**				
Dominant (kg)	22.40 ± 0.41	22.66 ± 0.39	23.05 ± 0.41	0.01
Non-dominant (kg)	20.26 ± 0.40	20.09 ± 0.38	20.45 ± 0.40	0.13
**Knee extension force:**				
Symptomatic leg (N)	356.01 ± 5.95	354.84 ± 5.32	358.61 ± 5.38	0.31
Non-symptomatic leg (N)	378.22 ± 5.84	378.00 ± 5.65	379.90 ± 5.79	0.45
**Physical performances**				
Gait speed (m/s)	0.96 ± 0.02	1.10 ± 0.02	1.14 ± 0.02	<0.001
TUGT (s)	9.81 ± 0.19	8.81 ± 0.20	8.65 ± 0.17	<0.001
STS (s)	14.87 ± 0.37	13.86 ± 0.35	13.28 ± 0.39	<0.001
6MWT (m)	371.22 ± 5.95	400.05 ± 6.32	421.20 ± 5.83	<0.001
